# Case of Tænia Solium Similating Pleuritis

**Published:** 1876-06

**Authors:** J. C. Beauchamp

**Affiliations:** Griffin, Ga.


### CASE OF TÆNIA SOLIUM SIMILATING PLEURITIS.

By J. C. BEAUCHAMP, M.D., of Griffin, Ga.

On the evening of the 14th inst. (April), I was called to see Mrs. S. She was taken with a chill, or rigor, about four hours previous to the time that I was called. Upon my arrival I found she had some fever; was suffering very much from pain in the right side; respiration labored and jerking; paroxysms of short, dry, hacking cough. Auscultation and percussion gave no indication of trouble in the lungs, neither of adhesion or effusion. Being somewhat at a loss as to the cause of the trouble, I attacked the most prominent symptoms, by administering an opiate, applying a blister to the side and an expectorant for the cough. Next day found the same symptoms, though in a modified degree. Learning that her bowels had not moved for two or three days, I ordered an enema to be given composed of half pint of warm water and a teaspoonful of salt. In a short time her bowels moved, and little else was voided besides three or four feet of tape-worm of very large size, of the class *tœnia solium.* The above were certainly the symptoms of acute pleuritis, but in a short time after the worm was voided the symptoms disappeared. A case similar to this is found in Watson's Practice, taken from the *Journal of Medicine and Surgery* of Paris, in 1844

